# Sister Chromatid Exchange and Genomic Instability in Soft Tissue Sarcomas: Potential Implications for Response to DNA-Damaging Treatments

**DOI:** 10.1155/2018/3082526

**Published:** 2018-05-07

**Authors:** Abdulazeez Salawu, Kristin Wright, Afnan Al-Kathiri, Lynda Wyld, Malcolm Reed, Karen Sisley

**Affiliations:** ^1^Medical School, University of Sheffield, Sheffield, UK; ^2^Faculty of Medical Sciences, Al Baha University, Al-Bahah, Saudi Arabia; ^3^Brighton and Sussex Medical School, University of Sussex, Brighton, UK

## Abstract

Sarcomas are rare heterogeneous malignancies of mesenchymal origin characterised by complex karyotypes but no specific abnormalities. Recurrence is common, and metastatic disease carries poor survival despite standard DNA-damaging radiotherapy or chemotherapy. DNA double-strand breaks (DSBs) are either repaired by mechanisms such as homologous recombination (HR) or result in cell death by apoptosis. Endogenous *γ*H2AX formation and SCE formation are early and late events, respectively, and their levels are considered surrogate measures of genomic instability. Combined *γ*H2AX and SCE analysis was used to evaluate endogenous DNA DSB levels (and their subsequent repair) in 9 primary sarcoma cell lines and compared with well-established commercial lines. All the sarcoma cell lines had elevated *γ*H2AX and SCE levels, but there was no correlation between the DNA DSB frequency and subsequent SCE. Typically, radioresistant osteosarcoma cells had relatively low *γ*H2AX frequency but high SCE counts suggestive of efficient DNA repair. Conversely, liposarcoma cells derived from a radiosensitive tumour had high H2AX but relatively lower SCE levels that may imply inefficient DNA DSB repair. To our knowledge, this is the first report that correlates H2AX and SCE levels in primary sarcoma cell lines and may provide insight into potential response to DNA-damaging treatments.

## 1. Introduction

Soft tissue sarcomas (STSs) are a group of rare, heterogeneous malignancies of mesenchymal origin that affect around 2300 people a year in the UK [1]. They comprise less than 1% of all cancer diagnoses among adults but pose a significant diagnostic challenge with over 22 different subtypes and over 100 distinct morphologies (ICD-10) [2]. Current treatment recommendations for localised disease mainly involve a combination of surgery and radiotherapy in most subtypes, with chemotherapy reserved for a few sensitive subtypes [3, 4]. Overall survival for localised disease is in the order of 50–60% at 5 years but with metastatic disease, and two-year survival is only 20–30% with a median survival of around 12 months with standard chemotherapy [5].

STSs fall into one of two large genomic classes: The first is characterised by known, specific abnormalities such as chromosomal translocations or gene mutations and comprises around 20% of STSs. The majority of sarcomas, on the other hand, are characterised by complex seemingly random DNA copy number aberrations across the entire genome. They have no known specific abnormalities (a notable exception is well-differentiated liposarcomas that frequently carry chromosome 12q amplification), and their complex karyotypes are believed to be the result of genomic instability, a hallmark of the cancer phenotype [6].

**Figure 1:**
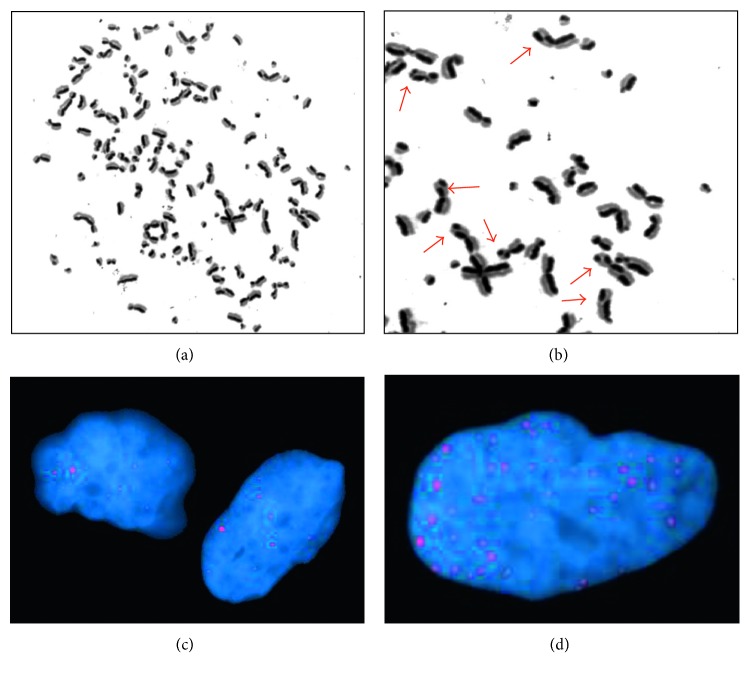
Endogenous sister chromatid exchanges and *γ*H2AX foci in sarcoma cells. (a) Harlequin-stained metaphase chromosomes from a Shef-*DDLPS* 02 (dedifferentiated liposarcoma) cell showing a hyperdiploid karyotype with over 120 chromosomes. (b) Higher magnification of a section of the same metaphase chromosome spread showing nine sister chromatid exchanges (red arrows). (c) Interphase nuclei of hTERT-RPE1 (human retinal epithelium) cells showing <10 endogenous *γ*H2AX foci each (red dots). (d) Shef-*DDLPS* 02 cell nucleus showing >10 *γ*H2AX foci (red dots). Metaphase chromosomes were stained with Hoechst 33258 dye followed by exposure to UV light. Interphase nuclei were stained using Cy3-conjugated rabbit anti-*γ*H2AX antibody and counterstained blue with DAPI.

Sister chromatid exchange (SCE) analysis is a method that allows the physical quantification of exchanged genetic material between sister chromatids during *in vitro* mitosis. Considered an endpoint of DNA repair by homologous recombination, measurement of SCE is a well-established and sensitive method for detecting DNA damage in the form of double-strand breaks (DSBs) induced by genotoxic agents [7–9]. The current method for SCE analysis developed by Perry and Wolff uses the thymidine substitute, 5′-bromo-2′-deoxyuridine (BrdU), which is incorporated into the DNA over two consecutive cell cycles creating an imbalance in the amount of BrdU in each sister chromatid [10]. Once stained, a harlequin banding pattern can be observed and the exchange of genetic material can be visualised as a mismatch between dark and lighter chromatids (Figure 1). The frequency of SCE can thus be enumerated in metaphase chromosome spreads prepared from cells toward the end of the second mitosis following BrdU treatment.

An even earlier event in the DNA DSB repair process is the phosphorylation of the histone molecule H2AX at serine 139 to *γ*H2AX, which accumulates at the sites of damage and recruits other DNA repair proteins. Phosphorylation of the histone protein H2AX is well recognised as an early step in the cellular recognition of DNA DSB for subsequent repair [11]. Using phospho-specific antibodies, *γ*H2AX foci at sites of DNA damage can be visualised and enumerated as another measure of DNA repair in eukaryotic cells [12]. Spontaneous SCE and *γ*H2AX formation has been observed in human cells where they are believed to be the result of endogenous DNA damage that causes collapsed replication forks and DNA double-stand breaks (DSBs) during mitosis. The frequency of endogenous DNA DSB repair has, therefore, been widely used as a surrogate measure of genomic instability in these cells [13, 14].

Unlike *γ*H2AX analysis which is done on interphase chromosomes, the specific timing requirement of metaphase spread preparation for SCE analysis makes this method technically challenging, and the majority of published studies utilising SCE analysis have focused on peripheral blood lymphocytes, which have relatively predictable doubling time. The observed frequency of endogenous SCE in normal human tissue and peripheral blood lymphocytes (PBLs) is around 6–8 per cell [15, 16]. Elevated frequency of SCE has been observed in PBL of patients with breast, prostate, gastric, ovarian, and cervical cancers showing up to three times the normal SCE frequency [13]. Individuals with Bloom's syndrome, a familial cancer-predisposing disease, also have remarkably high SCE frequency in their PBL [17], with an average of 89 per diploid cell [18]. Subsequent studies exploring the association between elevated PBL SCE frequency and cancer, however, showed mixed results and suggested a generalised increase in genetic instability but no specific links to tumour initiation or biomarker development [19–22].

SCE data on cancer cells are derived largely from haematologic malignancies, such as leukaemia, where tumour cells are prominent in the circulation [23] while only a handful of studies have been published based on established and commercially available solid organ tumour cell lines [13, 24–26]. With the exception of uveal melanomas [13], these have all shown that tumour cells possess elevated SCE frequency in keeping with genetic instability as a hallmark of cancer. Unfortunately, there is poor representation of STSs among commercially available cell lines, with the majority being translocation-driven subtypes. Furthermore, the limitations of commercial cell lines as an *in vitro* disease model are increasingly being recognised. Cellular adaptation with prolonged culture in artificial conditions and a lack of cellular heterogeneity that is characteristic of *in vivo* tumours are important factors that are believed to account for poor correlation of tumour cell line response with clinical outcomes. To ameliorate these important limitations, a widely accepted alternative to commercial cell lines is those directly derived from tumours (primary tumour cells) in early culture with less adaptation to artificial conditions. Ethical and logistic constraints associated with obtaining fresh tumour tissue (typically within minutes of surgery or biopsy), variable rates of successful establishment in culture and unpredictable subsequent *in vitro* behaviour make it rather challenging to utilise them for research in general and SCE analysis in particular.

In this study, we assessed endogenous genomic DNA damage/repair in a cohort of primary STS cell lines representing a range of subtypes. The frequency of early and late endogenous DNA DSB repair was measured using *γ*H2AX and SCE, respectively, as surrogates for genomic instability and compared to those observed in long-established, commercially available sarcoma cell lines, low SCE tumour cells, and nontumour cells.

## 2. Materials and Methods

### 2.1. Cell Lines and Cultures

Primary STS cell lines were developed from patient samples collected at the Royal Hallamshire Hospital, Sheffield, as previously described [27]. Informed consent was obtained from each patient after ethical approval (reference number 09/H1313/52), and tissue samples were handled in accordance with research ethics guidelines and the Human Tissue Act 2004. Short tandem repeat (STR) profiling was used to confirm the identity of all cell lines included in this study [27]. The sarcoma cell lines SOM-196b and hTERT-RPE1 were maintained in culture as previously described [13, 27] and subcultured as required.

### 2.2. Sister Chromatid Exchange Analysis

SCE analysis was performed as previously described [13]. Cell cultures were incubated with 0.24 *µ*M 5′-bromo-2′-deoxyuridine (BrdU) for approximately two cell cycles. This duration varied between 3 and 7 days depending on the proliferation rate of the cells, and multiple attempts were required to identify the required duration, particularly for the primary cell lines. Cell cycling was arrested in metaphase by addition of 10 *µ*g/ml colcemid and incubating for 2–4 hours (Thermo Fisher Scientific®, Paisley, UK). Chromosomes were then harvested by trypsinization and centrifuged and resuspended in 0.075 M potassium chloride (KCl) at 37°C for 40 minutes before fixing in a 3 : 1 methanol to acetic acid solution. Metaphase chromosome spreads were prepared on cold wet slides before staining with an adapted Perry and Wolff method with incubation in Hoechst 33258 dye for 15 minutes and exposure to UV-light for 12 minutes. Stained metaphases were visualised with a BH-2 light microscope, and images were captured with a Cohu® high-performance CCD camera (Cohu Electronics, San Diego CA, USA) and Powergene® software (Applied Imaging, Santa Clara, CA, USA). The number of chromosomes and exchanges in each metaphase spread was recorded. Chromosomes that were clearly overlapping or twisted were excluded from the exchange count, as is consistent with previous reports.

SCE counts were performed on up to 30 harlequin-stained metaphase chromosome spreads (Figure 1) where available. However, due to technical difficulties with the harlequin staining technique, it was not always possible to obtain a sufficient number of metaphase chromosome spreads from a single culture and chromosome harvest. In these cases, SCE analysis was repeated at a subsequent culture passage in order to obtain sufficient numbers (Table 1). For primary sarcoma cell lines, a minimum of ten analysed metaphase spreads was required for inclusion in this study.

### 2.3. *γ*H2AX Assay

Analysis of *γ*H2AX foci formation was again performed as previously described [13]. Viable cells were seeded at a density of 20,000 each on glass coverslips placed in six-well plates and cultured overnight. The cells were then washed with ice-cold phosphate-buffered saline (PBS) before fixing with 3% paraformaldehyde. They were then rinsed with PBS and permeabilised with 0.2% Triton-X for 5 minutes before blocking with 10% goat serum for 1 hour. Following another brief wash with PBS, the cells were incubated at 4°C overnight in the dark with Cy3-conjugated rabbit anti*γ*H2AX antibody (Cell Signaling Technologies®, Danvers, MA, USA) diluted 1 : 500 in 10% goat serum in PBS. Cells were subsequently washed on a shaker with PBS, and the coverslips were inverted onto microscope slides and mounted with Vectashield containing DAPI (Vector®, Peterborough, UK). Slides were stored in the dark at 4°C, and the number of foci per nucleus in 100 cells was counted on a UV spectrum-red fluorescent Nikon image analysis microscope at 100x magnification.

## 3. Results

### 3.1. Endogenous SCE Levels in Sarcoma Cell Lines

Nine primary cell lines, representing four soft tissue sarcoma subtypes, were analysed for SCE (Table 1). Two of these are morphologically distinct cultures that represent separate tumour cell clones (variants) derived from a single leiomyosarcoma, as we have previously demonstrated [27]. The four established and commercially available sarcoma cell lines U-2 OS, SK-LMS-1, SK-UT-1, and SW-1353, all of which are known to have complex karyotypes and high levels of endogenous SCE [13], were analysed as high-SCE controls. A nontumour cell line, hTERT-RPE1, and a uveal melanoma cell line, SOM-196b, previously shown to possess low SCE levels [13] were used as normal and low SCE controls, respectively.

Tumour cells were frequently hyperdiploid with significant inter- and intratumour heterogeneity in terms of chromosome number (Figure 1 and Table 1). To account for this heterogeneity and facilitate accurate comparison with normal diploid cells, the number of exchanges visualised per metaphase spread was normalised for a diploid (2*n*) karyotype by multiplying observed SCE by 46 and then dividing by the observed chromosome number.

### 3.2. Elevated Endogenous SCE Levels in Primary Sarcoma Cell Lines

**Figure 2:**
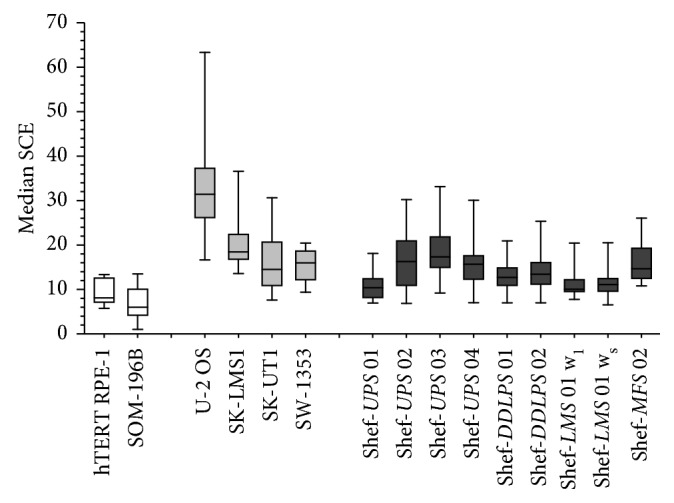
Frequency of sister chromatid exchange (SCE) in sarcoma cell lines. Normal and low SCE controls (clear boxes) had SCE frequency within the expected normal range (6–8 per 2*n* cell). All the sarcoma cell lines show SCE frequency above the normal range with primary sarcoma cells (dark grey boxes) comparable to established commercially available sarcoma cells (light grey boxes). SCE counts were normalised for diploid (2*n*) karyotype by multiplying the number of observed SCE by 46 and then dividing by the observed chromosome number. Boxes represent the interquartile range with horizontal line at median, while whiskers represent the minimum and maximum enumerated SCEs derived from 10 to 30 metaphase chromosome spreads.

The control cell lines SOM-196b and hTERT-RPE1 had median SCE counts of 6 and 8 per diploid (2*n*) metaphase spread, respectively, as expected (Figure 2). Certain factors are known to potentially confound the results of endogenous SCE analysis in cultured cells. For example, BrdU that is used to substitute thymidine residues in DNA and required for harlequin staining pattern of sister chromatids for analysis is known to be genotoxic and can result in slightly increased SCE frequency of around 1–3 per metaphase [28–30]. Furthermore, it has been demonstrated that certain media supplements (such as antibiotics or serum) can induce SCE in cultured cells *in vitro* [31, 32]. In order to minimise these effects, the lowest BrdU concentration (0.24M) that produced satisfactory banding was used in all experiments [13], and normal cell line controls were cultured using identical media and supplements as the primary sarcoma cells prior to SCE analysis. The normal control cell line demonstrated the SCE level that was within the expected range of 6–8 per metaphase spread, suggesting that these potential artefacts if present had minimal effect on our results.

All four commercially available sarcoma cell lines had endogenous SCE levels that were well above the expected normal range of 6–8, with median SCE counts of 15–31 per 2*n* metaphase spread (Table 1). Among these, it was notable that the osteosarcoma cell line U-2 OS had SCE levels (31 per 2*n* metaphase spread) that were nearly double those seen among the other sarcoma cell lines (Figure 2). SCE levels observed in the primary sarcoma cell lines were similar to those of the commercial lines. All nine primary sarcoma cell lines had SCE levels that were higher than the normal range with at least 7 endogenous SCEs seen within each metaphase chromosome spread and median SCE counts of between 10 and 18 per 2*n* metaphase spread. The elevated SCE levels were independent of chromosome number and STS subtype (Table 1 and Figure 2).

In the four primary sarcoma cell lines, where SCE analysis was repeated after approximately 4 months, the additional 5 to 15 culture passages did not appear to significantly affect the SCE levels. Furthermore, it was interesting to note that in Shef-*UPS* 02 and Shef-*UPS* 03, despite an increase in the median and range of the chromosome numbers observed between the two time points, their SCE frequency remained at about the same level when normalised for a diploid karyotype (Table 1).

### 3.3. Endogenous *γ*H2AX Foci Are Increased in Sarcoma Cells but Do Not Correlate with SCE Levels

**Figure 3:**
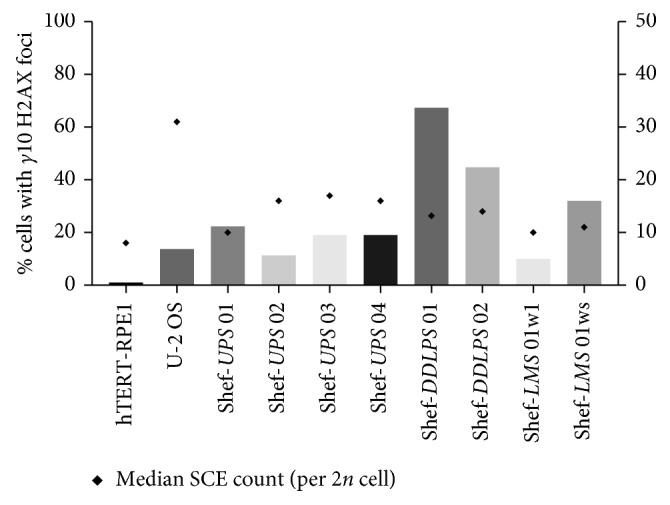
Endogenous *γ*H2AX foci and sister chromatid exchange analysis in sarcoma cell lines. No relationship was seen between the frequency of endogenous *γ*H2AX foci (bars) and SCE (corresponding black dots) among sarcoma cell lines (Spearman's *r*^2^=0.029; *p*=0.99). All nine cell lines however showed high frequency of endogenous *γ*H2AX foci and SCE, compared with nontumour control hTERT-RPE1 cells that had no cells with more than 10 endogenous *γ*H2AX foci. Bars plotted along the left *y*-axis represent the percentage of 100 randomly selected cells in each corresponding cell line with >10 endogenous *γ*H2AX foci, and data are shown as the mean of 3 independent repeat experiments. Black dots plotted along the right *y*-axis represent the median SCE counts from 10 to 30 metaphase chromosome spreads. The SCE counts were normalised for a diploid (2*n*) karyotype by multiplying the number of observed SCE by 46 and then dividing by the observed chromosome number.

Detection of phosphorylated H2AX (*γ*H2AX) foci by immunofluorescence was used for the detection of endogenous DNA double-strand breaks (DSBs). We performed *γ*H2AX analysis on eight primary STS cell lines. The osteosarcoma (U-2 OS) and retinal epithelial (hTERT-RPE1) cell lines were used as tumour and nontumour cell line controls, respectively. The results were correlated with the corresponding endogenous SCE levels and are summarised in Figure 3.

The normal cell line hTERT-RPE1 showed very little evidence of endogenous DNA DSB with no cells showing more than 10 *γ*H2AX foci. Among all the sarcoma cell lines, however, at least 10% of cells showed more than 10 *γ*H2AX foci. There was no relationship between the SCE levels and frequency of *γ*H2AX foci among the sarcoma cell lines (Spearman's *r*^2^=0.029; *p*=0.99). U-2 OS, the osteosarcoma cell line, that had almost double the number of SCEs seen among the other sarcoma cell lines surprisingly had relatively few *γ*H2AX foci (Figure 3). Conversely, the highest frequency of *γ*H2AX foci (>65% of cells showing >10 foci) was observed in dedifferentiated liposarcoma cell lines Shef-*DDLPS* 01 and Shef-*DDLPS* 02, whose SCE levels were only moderately elevated with medians of 13 and 14 per 2*n* metaphase spread, respectively (Table 1 and Figure 2). Interestingly, one of these two cell lines was derived from a dedifferentiated liposarcoma that prior to excision was treated with radiotherapy to which the patient had a significant radiologic and histologic response [27]. Another notable observation was the more than threefold difference in the frequency of cells showing >10 *γ*H2AX foci between w_1_ and w_s_ variants of Shef-*LMS* 01, which had very similar SCE levels (Figure 3).

## 4. Discussion

Reports of SCE analysis performed directly on solid tumour cells are rare in the published literature. Precise timing of BrdU exposure and chromosome harvest is largely unpredictable in many solid tumour cell lines, but especially so among primary cell cultures, such as those used in this study. To our knowledge, this is the first report of combined SCE and *γ*H2AX analysis for the measurement of genomic instability in sarcoma cells.

The nine primary tumour cell lines in this study have previously been shown to possess very complex karyotypes [27]. It was therefore not surprising that the endogenous SCE levels observed were consistently higher than the normal range even after correction for aneuploidy (Table 1 and Figure 2). Given the magnitude of genomic perturbation that is pervasive among these tumours, it was expected that the SCE frequencies would be even higher than those observed. However, when compared with other cancers, these results are concordant with previous studies that reported high SCE frequency in tumour cells obtained from the peripheral circulation in leukaemia patients as well as lymphoma, melanoma, breast, and colon cancer cell lines [23–26].

In support of the genomic instability suggested by their elevated SCE levels, *γ*H2AX analysis also showed high levels of endogenous DNA DSBs among all the sarcoma cell lines compared with normal control cells (Figure 3). These results are in line with those of Yu et al., as well as more recent studies that demonstrated elevated endogenous *γ*H2AX foci among 17 cancer cell lines from the NCI-60 panel with 20–95% of cells showing foci [33, 34]. In the same study, they also found that the number of foci seen correlated with the magnitude of karyotypic complexity of the cell lines evaluated, which supports our finding of high SCE levels among our cell lines that are derived from soft tissue sarcomas with very complex karyotypes. However, it does not account for the significant difference in the frequency of endogenous DNA DSBs observed between the two variants (w_s_ and w_1_) of the leiomyosarcoma cell line Shef-*LMS* 01 (Figure 3) even though they had very similar average SCE frequency and chromosome number (Figure 2 and Table 1). Endogenous *γ*H2AX foci are believed to represent DNA DSBs resulting from replication fork collapse/stress during the cell cycle [11], while SCE represents their subsequent repair. One possible explanation is that the cellular capacity for DNA repair by homologous recombination in these two tumour cell clones is finite resulting in their similar SCE levels despite the significant difference in the endogenous DSBs. The remaining DNA damage in the *w*_s_ variant may have been repaired via other cellular mechanisms such as nonhomologous end-joining (NHEJ), which is also preceded by *γ*H2AX formation [35, 36], or perhaps a relatively higher proportion of the cells were directed towards apoptosis. This hypothesis warrants further investigation to evaluate both endogenous and induced (in response to IR and other genotoxic agents) apoptosis and NHEJ among these cell lines.

Similarly, there was no correlation demonstrated between the frequency of SCE and endogenous H2AX foci among all the cell lines in this study when evaluated by Spearman's test. An illustrative example was the dedifferentiated liposarcoma cell line Shef-*DDLPS* 02 that had high endogenous *γ*H2AX foci but relatively low SCE counts (Figure 3). A possible explanation is that, despite the evidence of high frequency of DNA DSBs evidenced by the number of *γ*H2AX foci, the tumour cells have a poor ability to perform subsequent DNA repair by homologous recombination resulting in paradoxically low SCE frequency. This theory is supported by the good response (over 90% necrosis) to neoadjuvant radiotherapy that was seen on histologic examination of the parent tumour for this cell line (data not shown). Conversely, the U-2 OS cell line which had approximately double the SCE frequency observed in the other sarcoma cell lines had relatively few endogenous *γ*H2AX foci (Figure 3), an overall picture that would suggest highly efficient DNA repair. Given that U-2 OS is an osteosarcoma cell line and that this tumour subtype is known to be relatively resistant to radiotherapy, the results are not altogether surprising. Again, further studies to evaluate the *γ*H2AX foci, SCE counts, and apoptotic response of these cell lines to ionising radiation (IR) would be useful to further elucidate the impact of endogenous genomic instability on treatment response in these sarcomas.

Temporal changes in genomic instability with *in vitro* culture were assessed when SCE analysis of four primary cell lines was repeated after 4 months (Table 1). The results showed that the SCE frequency remained around the same level with time in culture, suggesting that the magnitude of genetic instability remained fairly constant in these primary sarcoma cells. However, according to the clonal evolution theory of cancer, longer duration in culture permits the accumulation of further genomic aberrations with time, and this is supported by an increase in the chromosome number observed in these cell lines with time and their acquisition of some new copy number abnormalities [27]. Perhaps, therefore, an alternative DNA repair mechanism with less accuracy than homologous recombination and not represented by SCE analysis is utilised by these tumour cells resulting in their continued accumulation of genomic aberrations. Once again, further longitudinal studies with evaluation of various alternative DNA repair mechanisms are warranted.

## 5. Conclusions

This study is the first of its kind in sarcomas and confirms that genomic instability is indeed characteristic of these tumours. It also highlights potential inter- and intratumour differences in DNA damage and/or responses as well as potential markers for the prediction of response to radiotherapy among these rare and heterogeneous tumours. Overall, the data, while not sufficient to make firm conclusions about their utility as biomarkers, suggest that there is still much to understand about the relationship between *γ*H2AX and SCE in sarcomas. Given that these tumours are so heterogeneous, it is likely that a number of explanations could exist including highly efficient homologous recombination, increased apoptosis, or alternative DNA repair pathways. Importantly, the stage is set for further studies that will improve our understanding of the mechanisms for genomic instability among sarcomas and how to utilise this for choosing appropriate treatments.

## Figures and Tables

**Table 1:** Endogenous sister chromatid exchange in sarcoma cell lines.

Cell line	Histological subtype	Passage number	Number of metaphases	Chromosome count, median (range)	Median SCE count		Range
					Observed	^a^Normalised
*Nontumour/low SCE controls*							
hTERT-RPE1	Normal retinal epithelium		7	43 (31–48)	8	8	6–13
SOM-196b	Uveal melanoma		11	45 (34–46)	6	6	1–14
*Commercially available sarcoma cell lines*							
U-2 OS	Osteosarcoma		30	71 (64–76)	47	31	17–63
SK-LMS-1	Leiomyosarcoma		10	100 (86–151)	42	18	14–37
SK-UT-1	Uterine leiomyosarcoma		20	44 (32–48)	14	15	8–31
SW-1353	Chondrosarcoma		12	48 (45–53)	17	16	9–20
*Primary sarcoma cell lines*							
Shef-*UPS* 01	Undifferentiated pleomorphic sarcoma	p69	22	59 (44–67)	13	10	7–18
^b^Shef-*UPS* 02	Undifferentiated pleomorphic sarcoma	p15	10	60 (56–60)	23	17	10–27
		p30	7	89 (53–114)	24	16	7–30
^b^Shef-*UPS* 03	Undifferentiated pleomorphic sarcoma	p17	29	56 (47–60)	23	18	9–33
		p33	24	71 (50–126)	23	17	9–32
^b^Shef-*UPS* 04	Undifferentiated pleomorphic sarcoma	p3	12	60 (51–65)	20	16	8–25
		p8	30	59 (52–69)	20	15	7–30
Shef-*DDLPS* 01	Dedifferentiated liposarcoma	p70	29	76 (41–97)	22	13	7–21
^b,c^Shef-*DDLPS* 02	Dedifferentiated liposarcoma	p12	30	108 (51–154)	35	14	9–25
		p23	29	117 (52–151)	33	13	7–26
Shef-*LMS* 01 w_1_	Leiomyosarcoma	p51	25	127 (74–152)	28	10	8–20
Shef-*LMS* 01 w_s_	Leiomyosarcoma	p62	22	118 (80–134)	31	11	7–21
Shef-*MFS* 02	Myxofibrosarcoma	p2	15	77 (42–105)	24	15	11–26

^a^Normalised for 2*n* karyotype by multiplying observed SCE counts by 46 and then dividing by observed chromosome number. ^b^Tumour was previously treated with radiotherapy prior to resection. ^c^SCE analysis was repeated after further *in vitro* culture.

## Data Availability

All the data presented in this study are available upon request by contacting the corresponding author.
